# RNA Sequencing Reveals the Expression Profiles of circRNAs and Indicates Hsa_circ_0070562 as a Pro-osteogenic Factor in Bone Marrow-Derived Mesenchymal Stem Cells of Patients With Ankylosing Spondylitis

**DOI:** 10.3389/fgene.2022.947120

**Published:** 2022-07-06

**Authors:** Shan Wang, Fenglei Chen, Chenying Zeng, Huimin Gu, Ziming Wang, Wenhui Yu, Yanfeng Wu, Huiyong Shen

**Affiliations:** ^1^ Center for Biotherapy, Eighth Affiliated Hospital of Sun Yat-sen University, Shenzhen, China; ^2^ Department of Orthopedics, Eighth Affiliated Hospital of Sun Yat-sen University, Shenzhen, China

**Keywords:** circRNAs, ankylosing spondylitis, BMSCs, Osteogenesis, circ_0070562

## Abstract

Recent studies have reported that circular RNAs (circRNAs) play a crucial regulatory role in a variety of human diseases. However, the roles of circRNAs in pathological osteogenesis in ankylosing spondylitis (AS) remain unclear. We conducted circRNA and miRNA expression profiling of osteogenically differentiated bone marrow-derived mesenchymal stem cells (BMSCs) of patients with AS compared with those of healthy donors (HDs) by RNA sequencing (RNA-seq). Results showed that a total of 31806 circRNAs were detected in the BMSC samples, of which 418 circRNAs were significantly differentially expressed (DE) with a fold change ≥2 and *p* value <0.05. Among these, 204 circRNAs were upregulated, and 214 were downregulated. GO and KEGG analyses demonstrated that the DE circRNAs were mainly involved in the regulation of biological processes of the cell matrix adhesion and the TGF-beta signaling pathway, which are closely related to AS. circRNA-miRNA interaction networks related to the TGF-beta signaling pathway were established. The results of qRT-PCR showed that has_circ_0070562 was significantly up-regulated in AS-MSCs. *In vitro* experiments showed that silencing of has_circ_0070562 weakened osteogenesis of AS-BMSCs. In conclusion, we identified numerous circRNAs that were dysregulated in AS-BMSCs compared with HD-BMSCs. Bioinformatic analyses suggested that these dysregulated circRNAs might play important functional roles in AS-BMSCs osteogenesis. Circ_0070562 functioned as a pro-ostegenic factor and might serve as a potential biomarker and a therapeutic target for AS.

## Introduction

Ankylosing spondylitis (AS) is a chronic autoimmune disease with high morbidity with a global incidence of approximately 2–5%. Chronic inflammation and pathologic osteogenesis are the core features of AS pathogenesis (Braun and Sieper, 2007; Sieper and Poddubnyy, 2017). When new bone forms in the sacroiliac joint, spine and the hip joint, the AS patients generally become ankylosed. Our previous studies documented that long noncoding RNAs and super enhancers are involved in the pathogenesis of osteogenesis in AS (Xie et al., 2016a; Yu et al., 2021). However, the mechanism of pathological osteogenesis needs to be further elucidated.

Bone marrow-derived mesenchymal stem cells (BMSCs), multipotent stem cells capable of immunoregulation and trilineage differentiation into osteoblasts, chondroblasts, and adipoblasts, play important roles in maintaining homeostasis *in vivo* (Keating, 2012). Additionally, BMSCs dysfunction has been demonstrated to be involved in the pathogenesis of several diseases (El-Jawhari et al., 2021). We previously reported that enhanced osteogenic differentiation of BMSCs from AS patients (AS-BMSCs) is related to pathological osteogenesis in AS, in which the aberrant TGF-beta signaling pathway and the imbalance between bone morphogenetic protein 2 (BMP2) and Noggin (NOG) represented pivotal mechanism of pathological osteogenesis in BMSCs of AS (Xie et al., 2016a; Xie et al., 2016b; Zheng et al., 2019). However, the specific mechanism of abnormal osteogenic differentiation, like the reason for the disorder of TGF-beta signaling pathway and imbalance between BMP2 and NOG in BMSCs from patients with AS remains to be elucidated.

In recent years, increasing evidence has indicated that alterations in circular RNA (circRNA) expression profiles are linked to a variety of disease processes, including AS progression (Kou et al., 2020; Li et al., 2021; Song et al., 2021; Tang et al., 2021; Wang et al., 2021; Zhang et al., 2022). CircRNAs, unlike canonically spliced linear RNAs, are single-stranded closed-loop RNA molecules lacking terminal 5′ caps and 3′ poly (A) tails that resist digestion by RNase and are relatively stable. They are enriched in miRNA-binding sites and can function as miRNA sponges or competing endogenous RNAs (ceRNAs) that naturally sequester and competitively inhibit miRNA activity. CircRNAs may also be central regulators of biological processes (Kristensen et al., 2019). Emerging studies have documented that some circRNAs are involved in osteogenesis by sponging miRNAs. For example, circRNA_33287 promotes the osteogenic differentiation of maxillary sinus membrane stem cells via miR-214–3p/Runx3 (Peng et al., 2019). However, few investigations have focused on the mechanisms of functional circRNAs in AS pathology, especially in the pathological osteogenesis of AS-BMSCs.

In this study, we used RNA-seq to examine the expression profiles of circRNAs and miRNAs in BMSCs from AS patients and HD after osteogenic induction. Parental genes of DE circRNAs were analysed by GO and KEGG. CircRNA-miRNA interaction networks were performed to predict the potential role of circRNAs in the process of AS. Then, qRT-PCR was used to further validate their expression. Lastly, we chose three circRNAs and designed small interfering RNAs to investigate their roles in the ostegenesis of HD- and AS-BMSCs. Our results revealed that circRNAs might play pivotal roles in pathologic osteogenesis. Hsa_circ_00070562 (circTET2) was up-regulated in AS-BMSCs and silencing it could obviously decrease osteogenesis of HD- and AS-BMSCs. Our study have provided new clues for studying the mechanisms underlying AS and hsa_circ_00070562, as a pro-ostegenic factor, might be a novel molecular target for the clinical diagnosis and treatment of AS.

## Materials and Methods

### Isolation and Culture of BMSCs

This study was approved by the Ethics Committee of the Eighth Affiliated Hospital of Sun Yat-sen University and conformed to the Ethical Guidelines of the Declaration of Helsinki. Twenty healthy donors (10 males, 10 females) between the ages of 20 and 30 years who had no history of any significant illness were selected as controls, and twenty AS patients (diagnosed according to the New York modified criteria (Linden et al., 1984); 10 males, 10 females) were recruited. All of the participants signed informed consent forms upon disclosure of the study details. BMSCs were isolated and cultured as described previously (Wu et al., 2011). Briefly, bone marrow was aspirated from ilium via puncturing. Then the BMSCs were precipitated, counted and seeded in the culturing dishes with 10% Fetal Bovine Serum (FBS, Sijiqing, China) in DMEM (Gibco) medium. After removing the supernatant and passaging, the BMSCs were attached and purified. BMSCs were used for the following experiments at passage 3.

### Flow Cytometry Phycoerythrin

BMSCs were digested with 0.25% trypsin supplemented with 0.53 mM EDTA (Gibco). After centrifugation, BMSCs were resuspended in phosphate-buffered saline (PBS) and incubated for 30 min with antibodies against human CD14-fluorescein isothiocyanate (FITC) 555397) and its FITC Mouse IgG2a, κ Isotype Control (554647); CD34-FITC (555821), and its FITC Mouse IgG1 κ Isotype Control (554679); CD45-APC (560973) and its APC Mouse IgG1 κ Isotype Control (554681); CD105-PE (560839) and its PE Mouse IgG1, κ Isotype Control (550617); CD90-PE (561970) and its PE Mouse IgG1, κ Isotype Control (550617); CD73-(FITC) 561254) and its FITC Mouse IgG1, κ Isotype Control 554679) or HLA-DR-PE (562304) and its PE Mouse IgG2a, κ Isotype Control 565363) (all from BD Biosciences, San Jose). Flow cytometry was performed using FACSCelesta (BD) to identify BMSCs phenotypes. FlowJo VX was used to analyze flow cytometry data.

### Osteogenic Differentiation

BMSCs were seeded in 12-well plates in osteogenic differentiation medium composed of DMEM, 10% FBS, 0.1 μM dexamethasone, 10 mM β-glycerol phosphate, 50 μM ascorbic acid, 100 IU/ml penicillin, and 100 IU/ml streptomycin (Sigma) for 14 days. The medium was replaced every 3 days.

### Alkaline Phosphatase (ALP) Activity Assay

BMSCs that had been osteogenically differentiated for 7 days were washed with PBS, fixed with 4% paraformaldehyde for 30 min, and then stained using an ALP staining kit (Jiancheng, Nanjing, A059-2) for 30 min, and images of the stained BMSCs were captured.

### Alizarin Red S Assay

BMSCs that had been osteogenically differentiated for 14 days were washed with PBS, fixed with 4% paraformaldehyde for 30 min, and then stained with 1% alizarin red S (Solarbio, G8550-25) for 15 min. The cells were washed to remove nonspecific staining, and images of the stained BMSCs were captured. For alizarin red S quantification, stained cells were destained with 10% cetylpyridinium chloride monohydrate (Sigma). After 1 h, absorbance was measured at 562 nm.

### Library Construction and circRNA-Sequencing

Total RNA was isolated from BMSCs using Magzol Reagent (Magen, China) according to the manufacturer’s protocol. The quantity of RNA was assessed using K5500 (Beijing Kaiao, China), and the integrity was determined with the Agilent 2,200 TapeStation (Agilent Technologies, United States ). rRNA was removed from the total RNA using a RiboCop rRNA Depletion Trial Kit (LEXOGEN, Australia). Then, the RNA was treated with RNase R (Epicentre, United States ) and fragmented into approximately 200 bp pieces. Subsequently, the purified RNA fragments were subjected to first strand and second strand cDNA synthesis followed by adaptor ligation and enrichment for a low cycle number according to the instructions of the NEBNext^®^ Ultra™ RNA Library Prep Kit for Illumina (NEB, United States ). The library products were evaluated using the Agilent 2,200 TapeStation and Qubit (Thermo Fisher Scientific, United States ) and then sequenced on the Illumina system (Illumina, United States ) with paired-end 150 bp reads by RiboBio Co., Ltd. (RiboBio, China).

### Identification and Quantification of circRNAs

After removing adapters with Trimmomatic tools (V0.36) (Bolger et al., 2014), the read quality was inspected using FastQC software (de Sena Brandine and Smith, 2019). The human reference genome hg19 (http://genome.ucsc.edu/) was used to map the reads. Two algorithms, CIRI2 (Gao et al., 2015) and CIRCexplorer2 (Dong et al., 2019), were used to detect circRNAs. If a circRNA was detected by both methods, it was considered an identified circRNA. The DESeq2 (Anders and Huber, 2010) package was used to identify DE circRNAs according to the following criteria: |log2 (fold change) | ≥1 and *p*-value < 0.05.

### GO and KEGG Pathway Analyses

GO functional annotations and KEGG pathway enrichment analyses were conducted using the clusterProfiler package in KOBAS 3.0 software (Bu et al., 2021). A *p*-value < 0.05 was used as the threshold of significant enrichment of the gene sets in KEGG enrichment analysis.

### Bioinformatics Analysis of circRNA-miRNA Regulatory Networks

TargetScan (v7.0) (Agarwal et al., 2015), RNAhybrid (Rehmsmeier et al., 2004) and miRanda (v3.3a) (Enright et al., 2003; John et al., 2004) were used to identify the potential miRNA targets of circRNAs. The construction of circRNA-miRNA networks was performed using Cytoscape (v3.1.0) software (Shannon et al., 2003).

### Real-Time Reverse Transcription PCR

TRIzol RNA isolation reagent (Invitrogen, Carlsbad, CA) was used to isolate total RNA from BMSCs. A NanoPhotometer N60 Touch (Implen, Germany) was used to measure RNA integrity and concentration. For circRNAs, reverse transcription was conducted using an Evo M-MLV reverse transcription kit (containing random primers) (Accurate Biology, China). Specific divergent primers spanning the back-splice junction sites of circRNAs were designed. For miRNA assays, the reverse primer (5′CAG​TGC​GTG​TCG​TGG​AGT 3′) specific to miRNAs was provided in the Mir-X miRNA First-Strand Synthesis Kit (TaKaRa, United States ), and cDNA was synthesized according to the manufacturer’s instructions. The primers shown in Supplementary Table S1 were utilized for the qPCR. The SYBR Green Pro Taq HS qPCR Mix kit (Accurate Biology, China) was used to perform all qPCR reactions. GAPDH and U6 was an internal control. The cycling parameters were as follows: 30 s at 95°C followed by 40 cycles of 5 s at 95°C and 20 s at 60 °C.

### DNA Agarose Gel Electrophoresis

2 μL of PCR product mixed with six x DNA loading buffer (D1010, Solarbio) was conducted to the 2% agarose gel electrophoresis. 3 μL of DM2000 marker (CW0632S, CWBIO) per run was used. After 30 min of electrophoresis, the gel was observed under the UV-light cross-linker (BIOLINK DNA,ANALYTIKJENA, Germany).

### RNase R Treatment

10^5^ of BMSCs were collected and total RNAs were exacted. 2 μg of RNAs were incubated with 3 U of RNase R (R0301, Geneseed, China) for 15 min at 37 C. After treatment with or RNase R, the RNA expression levels of circRNAs and other mRNAs were analyzed by RT-PCR and DNA agarose gel electrophoresis.

### Nuclear and Cytoplasmic Extraction

Cytoplasmic and nuclear fractions were isolated using the reagents in PARIS kit (AM 1921, Thermo Fisher Scientific) according to the manufacturer’s introductions. Briefly, BMSCs were lysed in the cell fraction buffer on ice for 20 min. After centrifugation at 300 *g* for 5 min at 4 C, the supernatant was collected as cytoplasmic fraction. Then the pellet was washed with nuclear fraction buffer on ice for 1 h. After centrifugation at 300 *g* for 5 min at 4 C again, the supernatant was collected as nuclear fraction. Then the expression of circRNAs were detected using qPCR.

### RNA Interference

Small interfering RNAs (siRNAs) targeting hsa_circ_0000348 (5′-3′AGA​CGA​CGA​GAG​UGG​AUGGAAdTdT), hsa_circ_0001493 (5′-3′UUGCCUAAAAUGUUGGCUCAAdTdT), hsa_circ_0070562 (5′-3′UUGCAGAUGUGUAGAAUUCAAdTdT) and the negative control (NC) (5′-3′UUCUCCGAACGUGUCACGUdTdT) were purchased from Accurate Biology (Guangzhou, China). When the seeded BMSCs reached 70–90% confluence, transfections were performed by using Opti‐MEM and Lipofectamine RNAiMAX (Thermo Fisher) according to the directions. Then, the BMSCs were collected and RNAs were exacted to measure the interference efficiency after 48 h or directly used in other experiments.

### Statistical Analysis

T tests and one-way analyses of variance followed by Bonferroni tests and Pearson correlation tests were performed in SPSS software. All data are expressed as means ± SEMs. *p* values <0.05 were considered to indicate a statistically significant difference. All circRNAs were named according to the circbase database. Novel circRNAs were named circ_gene.

## Results

### The Osteogenic Differentiation Capacity of AS-BMSCs Is Higher Than That of HD-BMSCs

**FIGURE 1:**
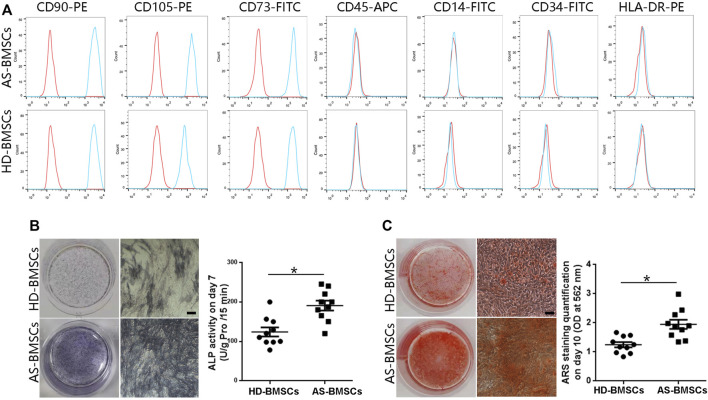
Immunophenotypes and osteogenic capacities of HD-BMSCs and AS-BMSCs. **(A)**. HD-BMSCs (*n* = 10) and AS-BMSCs (*n* = 10) were both positive for CD90, CD105, and CD73 and negative for CD45, CD14, CD34 and HLA-DR. **(B)**. Results of the alkaline phosphatase (ALP) assay for HD-BMSCs and AS-BMSCs on day 7 of osteogenic induction. Scale bar = 50 μm. The quantitative ALP assay results are shown on the right. **(C)**. Alizarin red S (ARS) staining of HD-BMSCs and AS-BMSCs on day 10 of osteogenic induction. Scale bar = 50 μm. The quantitative ARS staining results are shown on the right. The data are presented as the means ± SEMs. N = 10 per group. *p < 0.05.

Both HD-BMSCs and AS-BMSCs expressed typical MSC surface markers, as they were positive for CD90, CD73, and CD105 and negative for CD14, CD34, CD45, and HLA-DR (Figure 1A). The potential for osteogenic differentiation was confirmed following standard protocols. Alkaline phosphatase (ALP) assay and alizarin red S (ARS) staining showed that ALP activity and calcium deposition were much greater in osteogenically differentiated AS-BMSCs than in HD-BMSCs (Figures 1B, C), indicating that the osteogenic differentiation potential of AS-BMSCs was higher than that of HD-BMSCs, consistent with previous conclusion (Xie et al., 2016b; Liu et al., 2019; Zheng et al., 2019).

### Differential Expression Profiles of circRNAs in Osteogenically Differentiated AS-BMSCs and HD-BMSCs by RNA-Seq

**FIGURE 2:**
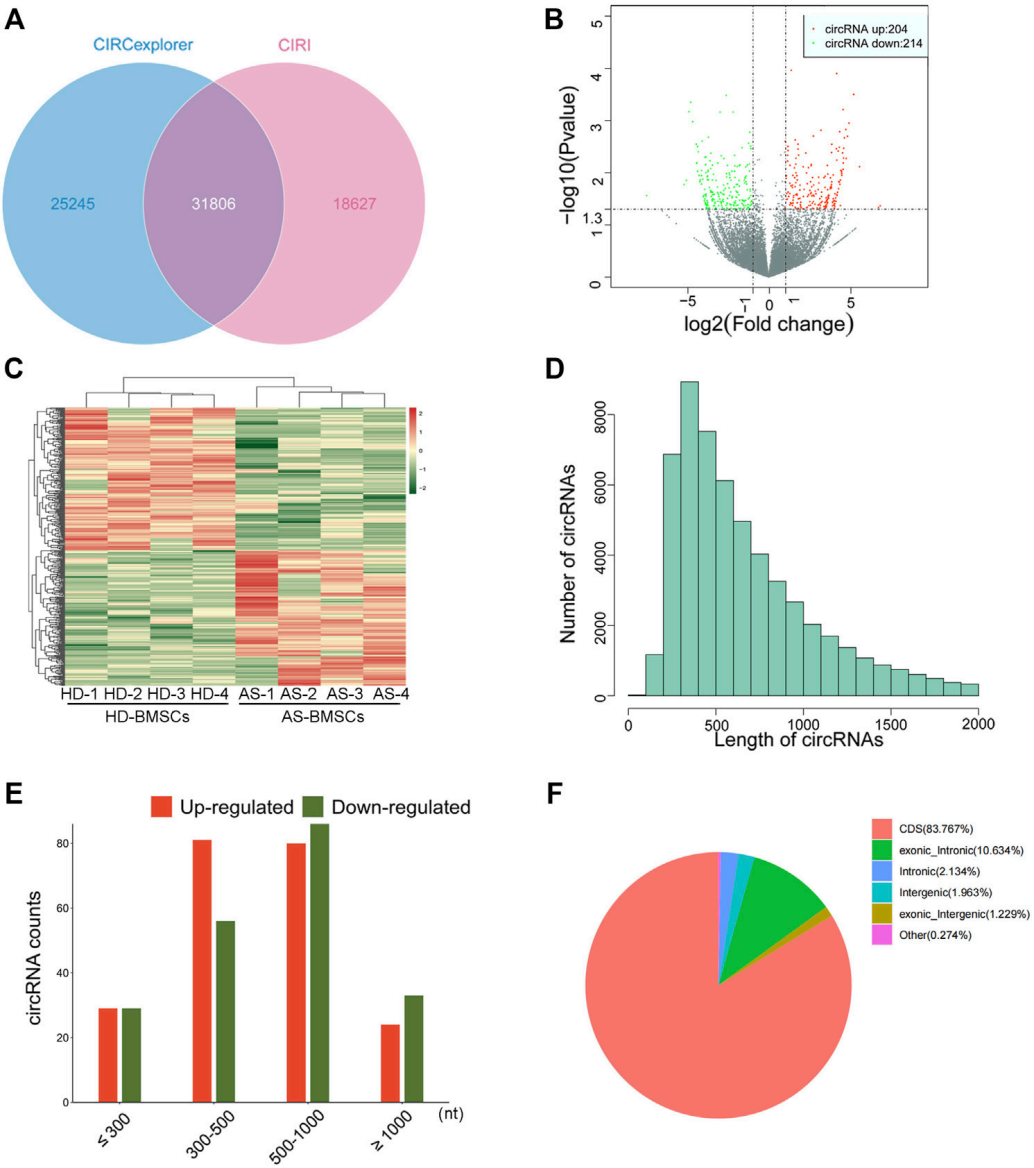
Differential expression profiles of circRNAs in HD-BMSCs and AS-BMSCs identified by RNAseq. **(A)**. Venn diagram showing circRNAs identified by both CIRI2 and CIRCexplorer2. **(B)**. Volcano plots of DE circRNAs in HD-BMSCs and AS-BMSCs. The vertical line indicates a 2.0-fold (log2 scale) change; the horizontal line represents the *p* value of 0.05 (-log10 scale). Red dots indicate the upregulated circRNAs. Green dots indicate the downregulated circRNAs. **(C)**. Heatmap of distinguishable and clustered circRNAs in HD-BMSCs and AS-BMSCs. **(D)**. The number of circRNAs of different lengths (nt). **(E)**. Number of upregulated and downregulated circRNAs based on nucleotide length (nt). **(F)**. Classification of the identified circRNAs by CIRI2 based on genomic origin.

Four pairs of AS-BMSCs and HD-BMSCs after ostegenic induction for 7 days were subjected to circRNA sequencing. A total of 31806 circRNAs were detected by two algorithms of CIRI2 and CIRCexplorer2 in the two groups of samples (Figure 2A). The distribution of the circRNAs on human chromosomes is depicted (Supplementary Figure S1A). Approximately 99% of the identified circRNAs had abundances of <100 back-spliced reads (Supplementary Figure S1B). The volcano plot and scatter plot show the differences in circRNA expression between the two groups (Figure 2B; Supplementary Figure S1C). Among the 31806 identified circRNAs, 418 were differentially expressed with a fold-change of 2.0 and *p* value <0.05 (Supplementary Material Data Sheet 1). Among these, 204 circRNAs were upregulated, while 214 circRNAs were downregulated. The details of the top 20 circRNAs showing significant upregulation or downregulation by log2FC are shown in Table 1. Among the 418 DE circRNAs, 148 circRNAs were identified as new circRNAs that had not been annotated in the circBase or circ2Traits database. In addition, hierarchical clustering revealed that circRNA expression levels were distinguishable, as shown in Figure 2C. Approximately 98% of the identified circRNAs were <2,000 nucleotides (nt) in length (Figure 2D), and the length of most differentially expressed circRNAs with statistical significance between the two groups was also <2,000 nt (Figure 2E). In addition, most circRNAs were transcribed from protein-coding exons, some were from introns, and a few were intergenic (Figure 2F). All of the differentially expressed circRNAs in AS were located in the exonic region based on the sequence structure origin of the circRNAs. Summarily, circRNA sequencing demonstrated that the expression levels of some circRNAs in BMSCs were significantly changed between patients with AS and healthy donors.

**TABLE 1:** Top 20 dysregulated circRNAs in AS-BMSCs compared with HD-BMSCs.

circName	Log_2_FC	*p* Value	Regulation	circBase ID	Gene name
chr2:202410266–202440049	6.85334	0.04316	up	NA	ALS2CR11
chr7:74119496–74133260	6.74553	0.04658	up	hsa_circ_0005513	GTF2I
chr13:100959446–101020828	5.58569	0.00765	up	hsa_circ_0030767	PCCA
chr16:532525–546859	5.22562	0.00032	up	hsa_circ_0002774	RAB11FIP3
chr13:21148519–21166543	4.93617	0.00112	up	NA	IFT88
chr11:57563049–57564464	4.84732	0.00200	up	NA	CTNND1,TMX2-CTNND1
chr1:203151859–203152919	4.76498	0.00532	up	NA	CHI3L1
chr1:213037067–213061936	4.74656	0.00148	up	hsa_circ_0016408	FLVCR1
chr22:30050646–30064435	4.65577	0.00214	up	hsa_circ_0062778	NF2
chr17:45479498–45492285	6.85334	0.00339	up	hsa_circ_0000778	EFCAB13
chr5:65284463–65310553	-7.46996	0.02734	down	hsa_circ_0001493	ERBIN
chr19:8533658–8539128	-5.17427	0.01691	down	hsa_circ_0003765	HNRNPM
chr17:79254409–79258695	-5.04423	0.01406	down	hsa_circ_0005389	SLC38A10
chr10:127500783–127505094	-4.87062	0.00068	down	hsa_circ_0008369	UROS
chr3:171360630–171395484	-4.77575	0.00044	down	NA	PLD1
chr1:28116073–28120143	-4.65193	0.00105	down	hsa_circ_0008126	STX12
chr5:94134718–94259726	-4.42706	0.00283	down	hsa_circ_0073381	MCTP1
chr20:13509080–13561628	-4.40856	0.00656	down	hsa_circ_0059469	TASP1
chr1:103477969–103488552	-4.39007	0.00641	down	NA	COL11A1
chr13:21987791–21999817	-4.35586	0.00315	down	hsa_circ_0006732	ZDHHC20

### GO and KEGG Analyses

**FIGURE 3:**
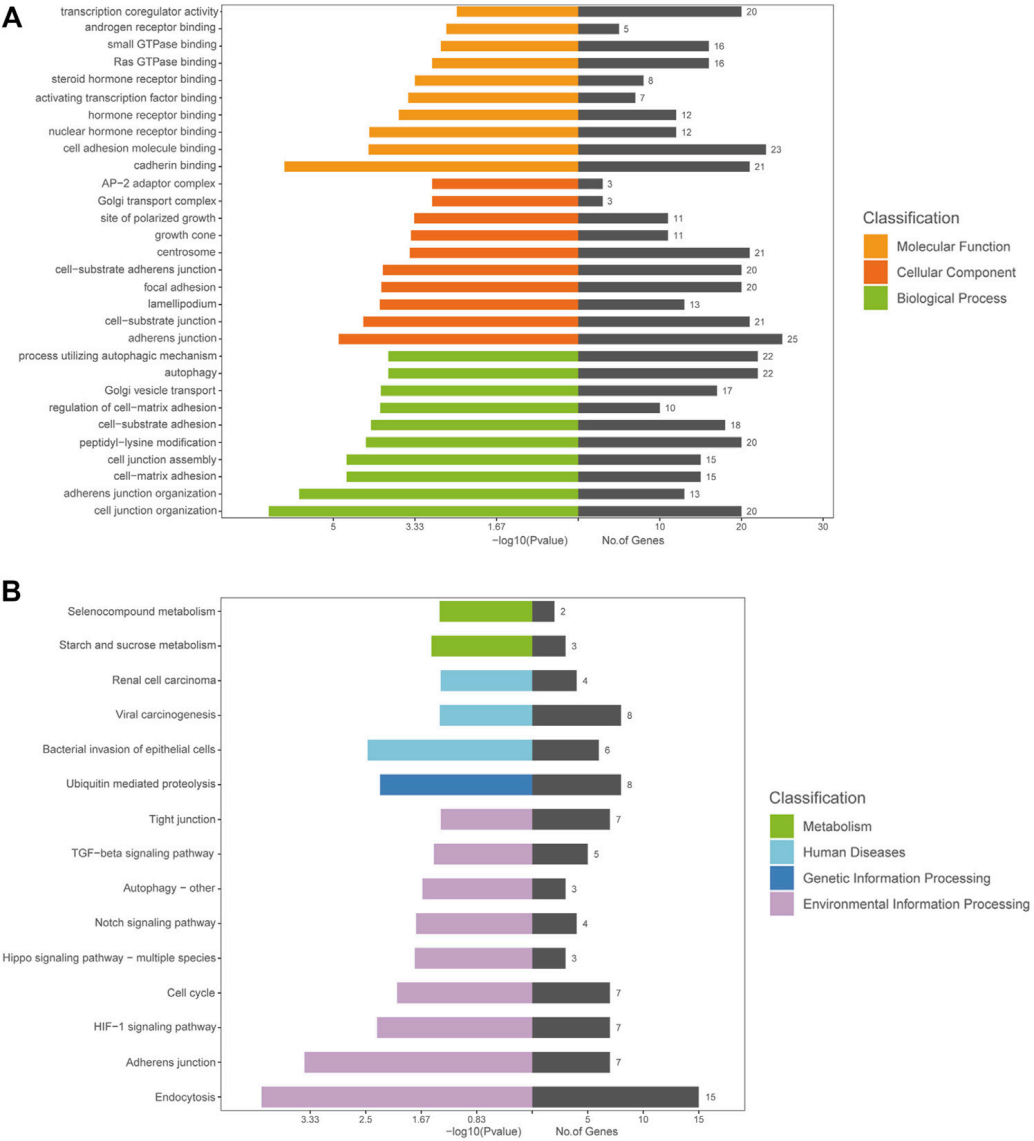
GO and KEGG analyses of the host genes of DE circRNAs in AS-BMSCs. **(A)**. GO annotations of the host genes of DE circRNAs. The left bar plot presents the enrichment scores [−log_10_ (*p* value)] of the top 10 significantly enriched GO terms in the biological processes, cellular components and molecular functions categories. The bar plot on the right presents the number of genes enriched in each term. **(B)**. KEGG analysis of the host genes of DE circRNAs.

DE circRNAs and their parental genes were further analyzed based on the Gene Ontology (GO) and Kyoto Encyclopedia of Genes and Genomes (KEGG) databases to predict circRNA functions and molecular interactions among genes. The top 10 enriched GO terms for biological process (BP), cellular component (CC), and molecular function (MF) are shown in Figure 3A; Supplementary Tables S2–4. The most significantly enriched GO terms in the BP, CC and MF categories were cell junction organization, adherens junction and cadherin binding, respectively. The top 15 enriched and meaningful pathways were related to endocytosis, the Hippo signaling pathway, autophagy, starch and sucrose metabolism and the TGF-beta signaling pathway (Figure 3B). The top 15 pathways and DE parental circRNAs associated with these pathways are shown in Table 2.

**TABLE 2:** The Top 15 Pathways with the Largest Significant Difference in KEGG Analysis of Parental Genes of DE circRNAs.

Term	ID	Pvalue	Count	geneNames
Endocytosis	hsa04144	0.00009	15	ACAP2/AP2A2/ARFGEF2/ARPC2/CAPZB/CBL/EHD2/KIF5B/MDM2/MVB12B/PARD3/PLD1/RAB11FIP3/SH3GLB1/WWP1
Adherens junction	hsa04520	0.00038	7	CREBBP/CTNNA1/CTNND1/EP300/NECTIN3/PARD3/PTPRM
Bacterial invasion of epithelial cells	hsa05100	0.00338	6	ARHGAP10/ARPC2/CBL/CTNNA1/CTTN/PTK2
HIF-1 signaling pathway	hsa04066	0.00468	7	CREBBP/CUL2/EP300/HK1/IFNGR2/IL6R/PFKP
Ubiquitin mediated proteolysis	hsa04120	0.00519	8	BIRC6/CBL/CUL2/MDM2/MID1/TRIM37/UBE2Z/WWP1
Cell cycle	hsa04110	0.00935	7	ATM/CDC14B/CREBBP/EP300/MDM2/RBL1/STAG1
Hippo signaling pathway - multiple species	hsa04392	0.01726	3	LATS1/NF2/YAP1
Notch signaling pathway	hsa04330	0.01803	4	CREBBP/EP300/NCOR2/NUMB
Autophagy - other	hsa04136	0.02247	3	ATG3/ATG4B/WIPI1
Starch and sucrose metabolism	hsa00500	0.03062	3	GBE1/HK1/PYGL
TGF-beta signaling pathway	hsa04350	0.03337	5	CREBBP/EP300/LTBP1/NEO1/RBL1
Selenocompound metabolism	hsa00450	0.04069	2	CTH/PAPSS1
Viral carcinogenesis	hsa05203	0.04097	8	CREBBP/EP300/GTF2E2/HDAC4/IL6ST/MDM2/RBL1/TBPL1
Renal cell carcinoma	hsa05211	0.04222	4	CREBBP/CUL2/EP300/FH
Tight junction	hsa04530	0.04245	7	ARPC2/BVES/CTTN/MPDZ/NF2/PARD3/TJP2

### Analyses of circRNA-miRNA Interaction in AS-BMSCs

**FIGURE 4:**
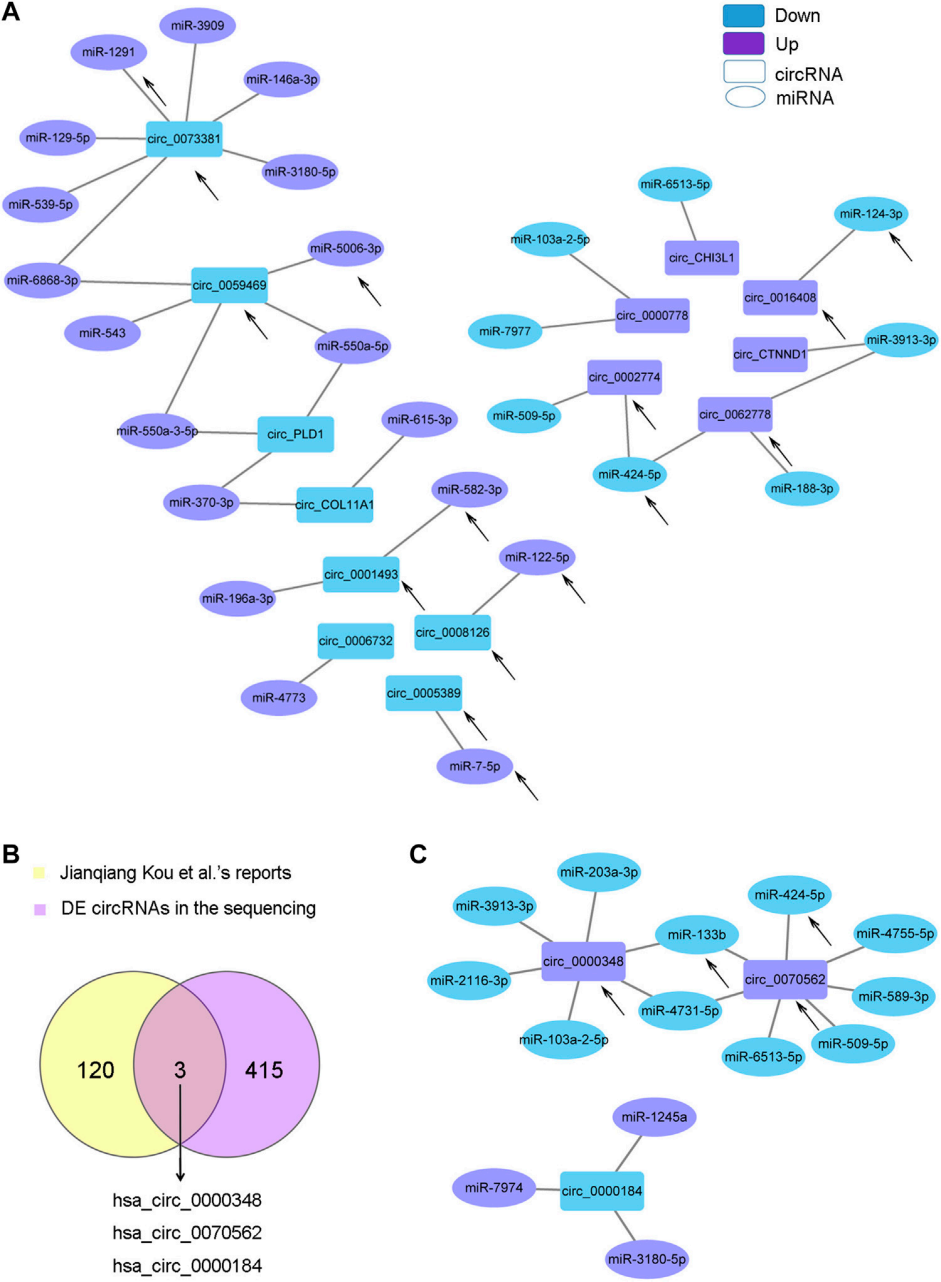
CircRNA-miRNA network. **(A)**. The circRNA-miRNA network based on the negative correlation of the top 20 DE circRNAs with DE miRNAs. **(B)**. Venn diagram showing the three key circRNAs identified by both of our circRNA sequencing and another team. **(C)**. The circRNA-miRNA network based on the negative correlation of the three circRNAs in **(B)** with DE miRNAs. The circles indicate miRNAs, and the rectangles indicate circRNAs. Purple nodes indicate upregulated genes, and blue nodes indicate downregulated genes (AS/HD). The edges indicate the interaction between two nodes. The black arrows indicate the key RNAs.

CircRNAs can act as sponge molecules to regulate the expression of genes by adsorbing miRNAs. We conducted miRNA expression profiling of the same samples used for circRNA sequencing and filtered 107 DE miRNAs (Supplementary Figure S2A and Supplementary Material Data Sheet 2). The potential target miRNAs of the top 20 dysregulated circRNAs were theoretically predicted according to conserved binding sites using miRanda, RNAhybrid and TargetScan analyses and intersected with the DE miRNAs (Supplementary Material Data Sheet 3). The matching circRNA-miRNA pairs with up-down or down-up relationships were constructed with Cytoscape (Figure 4A). 14 of the 20 circRNAs had complementary miRNA response elements. circRNA_0073381 and circRNA_0062778 were the two circRNAs with the largest number of predicted miRNA-binding sites among downregulated circRNAs and upregulated circRNAs, respectively (Figure 4A).

Jianqiang Kou et al. profiled the DE circRNAs in spinal ligament tissues from patients with AS compared with those from non-AS controls in 2020 (Kou et al., 2020). We intersected the DE circRNAs identified in Jianqiang Kou et al.‘s reports with our sequencing results and obtained three important circRNAs (two upregulated: circ_0000348 and circ_0070562, one downregulated: circ_0000184, Figure 4B). These three circRNAs may play important roles in AS. The matching pairs of these three circRNAs and their target miRNAs predicted in DE miRNAs are shown in Figure 4C.

**FIGURE 5:**
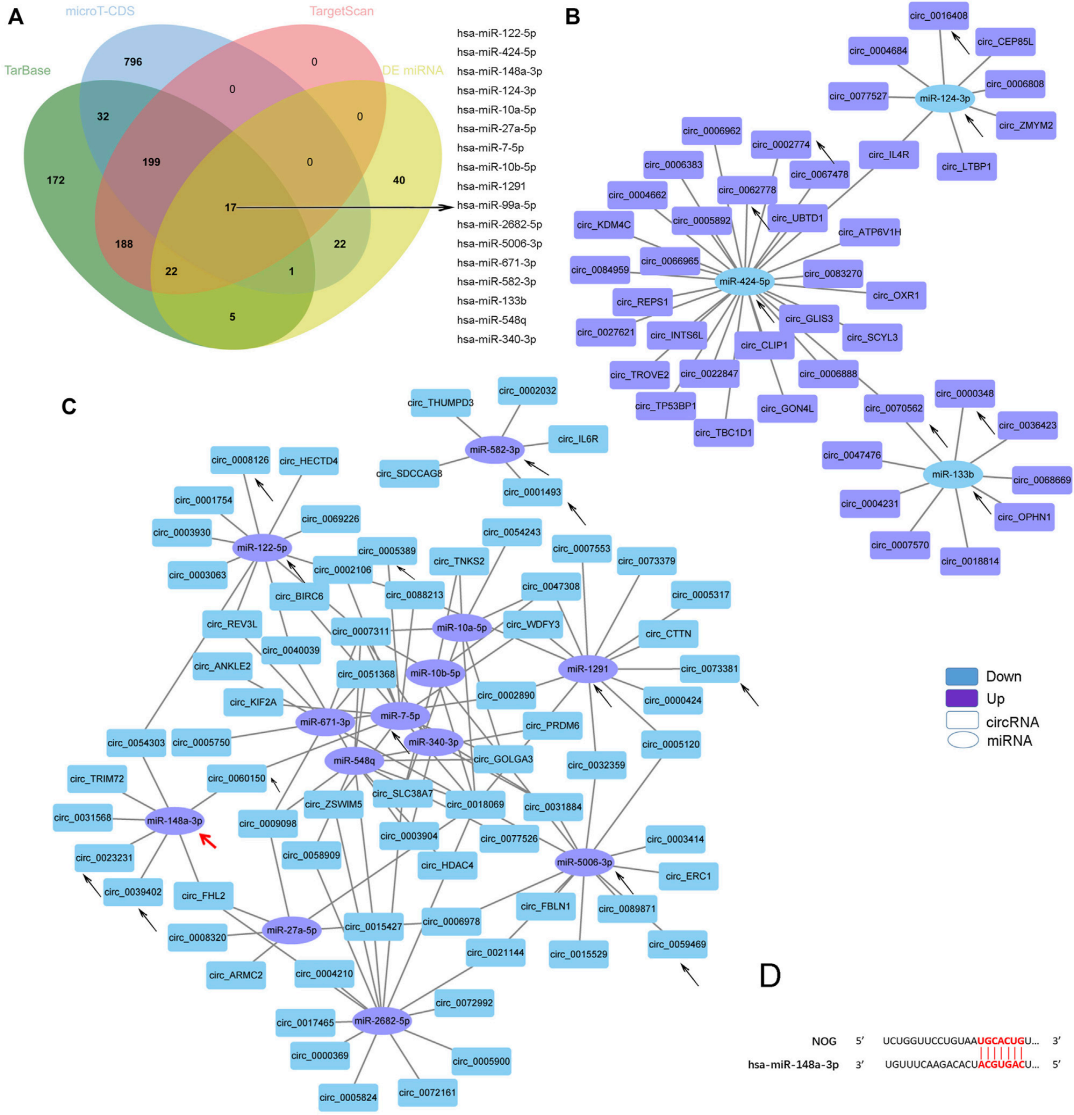
Predicted TGF-beta signaling pathway-related circRNA-miRNA network. **(A)**. Venn diagram showing the 17 DE miRNAs in AS-BMSCs compared with HD-BMSCs involved in the TGF-beta signaling pathway, as predicted by TarBase, micro-CDS and TargetScan. **(B)**. Network of circRNAs and miRNAs with up-down relationships potentially associated with the TGF-beta signaling pathway. **(C)**. Network of circRNAs and miRNAs with down-up relationships potentially associated with the TGF-beta signaling pathway. The circles indicate miRNAs, and the rectangles indicate circRNAs. Purple nodes indicate upregulated genes, and blue nodes indicate downregulated genes (AS/HD). The edges indicate the interaction between two nodes. The black arrows indicate the key RNAs. The red arrow indictes the miR-148a-3p that regulates NOG. **(D)**. Binding sites between miR-148a-3p and NOG were predicted by miRDB and TargetScan.

As miRNA sponges, the functions of circRNAs can be embodied in DE miRNAs. KEGG analysis of the DE miRNAs identified by our sequencing revealed that the miRNAs were enriched for several osteogenesis-related pathways, such as the MAPK, Hippo, FoxO, AMPK, Wnt, TGF-beta, and Hedgehog signaling pathways (Supplementary Figure S2B). As mentioned before, TGF-beta signaling pathway was reported to mediate the enhanced ostegenesis of AS-BMSCs (Xie et al., 2016a; Xie et al., 2016b; Zheng et al., 2019), so we screened miRNAs associated with the TGF-beta signaling pathway using the DIANA-miRPath v.3 platform (Reverse Search module) (Kiriakidou et al., 2004) through Tarbase, microT-CDS and TargetScan referring to the method used by Li et al. (Li et al., 2018). Based on analyses combining the results from the above three methods and the DE miRNAs, 16 miRNAs were identified (Figure 5A). A negative correlation between the transcript levels of these 16 miRNAs and DE circRNAs predicted potential interactions, which were constructed using Cytoscape (Figures 5B, C). A total of 162 pairs were predicted (Supplementary Material Data Sheet 4). Circ_0000348 and circ_0070562 were predicted to be involved in the TGF-beta signaling pathway by interacting with miR-133b. On the other hand, miR-148a-3p was predicted to regulate NOG (Figure 5D), a key cytokine that induces abnormal osteogenic differentiation in AS-BMSCs(Xie et al., 2016b). circ_0023231, circ_0031568, circ_0039402, circ_0054303, and circ_0060150 and two novel circRNAs, circ_TRIM72 and circ_ARMC56, were predicted to interact with miR-148a-3p with a negative correlation. The black arrows in Figures 4A, C; Figures 5B, C indicated the miRNAs involved in the TGF-beta signaling pathway and the interacting circRNAs from the top 20 dysregulated circRNAs.

### Validation of circRNA Expression and the Circ-miRNA Network

**FIGURE 6:**
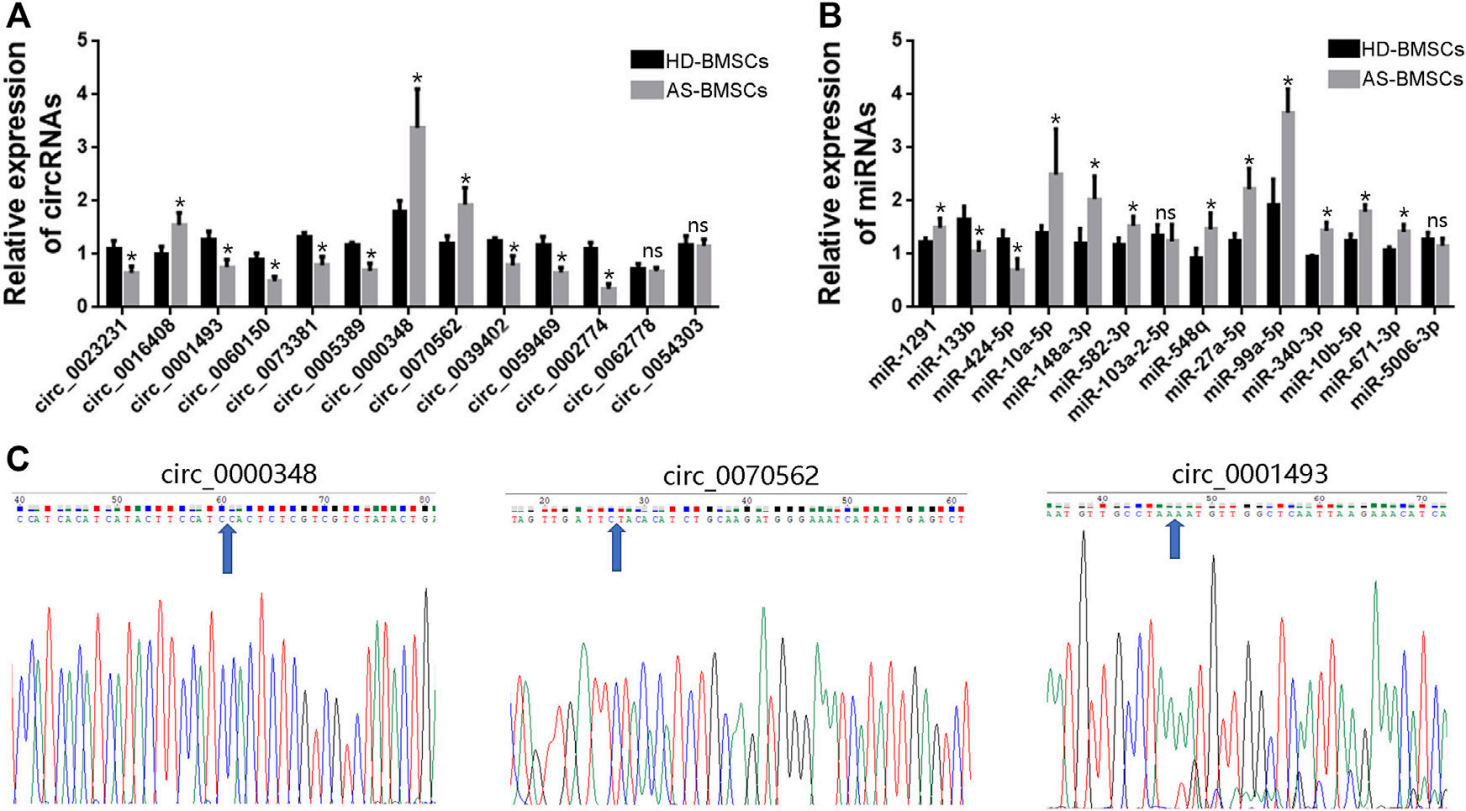
qRT-PCR validation of DE circRNAs and miRNAs. **(A)**. The relative expression levels of circRNAs involved in TGF-beta signaling pathway (the indicated circRNAs with black arrows) in 10 pairs of AS-BMSCs and HD-BMSCs. **(B)**. The relative expression levels of miRNAs selected from the DE miRNAs in 10 pairs of AS-BMSCs and HD-BMSCs. Data are presented as the means ± SEM. *p < 0.05. ns: not significant. N = 10 BMSCs per group. **(C)**. Sanger sequencing confirmed the back-splice junction sites of circ_0000348, circ_0070562 and circ_0001493. Blue arrowheads indicated the junction sites.

To verify the RNA-Seq data and the predicted analyses, qRT-PCR was performed for fourteen circRNAs and fourteen miRNAs selected from the above predicted analysis. The expression levels of those circRNAs and miRNAs were measured in 10 pairs of AS-BMSCs and HD-BMSCs after osteogenic induction for 7 days. We designed specific divergent primers spanning the back-splice junction sites of circRNAs. The results showed that the expression of circ_0000348, circ_0070562 and circ_0016408 was up-regulated and that the expression of circ_0001493, circ_0023231, circ_0060150, circ_0073381, circ_0005389, circ_0039402, circ_0059469 and circ_0002774 was down-regulated in osteogenically differentiated AS-BMSCs compared with HD-BMSCs (Figure 6A). And the expression of miR-148a-3p, miR-1291, miR-10a-5p, miR-582–3p, miR-548q, miR-27a-5p, miR-99A-5p, miR-340–3p, miR-10b-5p and miR-671–3p was increased, and the miR-133b and miR-424–5p was decreased in osteogenically differentiated AS-BMSCs compared with HD-BMSCs (Figure 6B). Based on the above results, the predicted networks of circ_0001493 with miR-582–3p, circ_0000348 and circ_0070562 with miR-133b, and circ_0070562 with miR-424–5p were validated to accord with the up-down or down-up relationships of circRNA and miRNA by qPCR. Moreover, the Sanger sequencing results of qRT-PCR amplified products of three circRNAs, including circ_0000348, circ_0070562 and circ_0001493 confirmed the back-splice junction sites (Figure 6C).

### Characterization of hsa_circ_0000348, hsa_circ_0001493 and hsa_circ_0070562 in BMSCs

**FIGURE 7:**
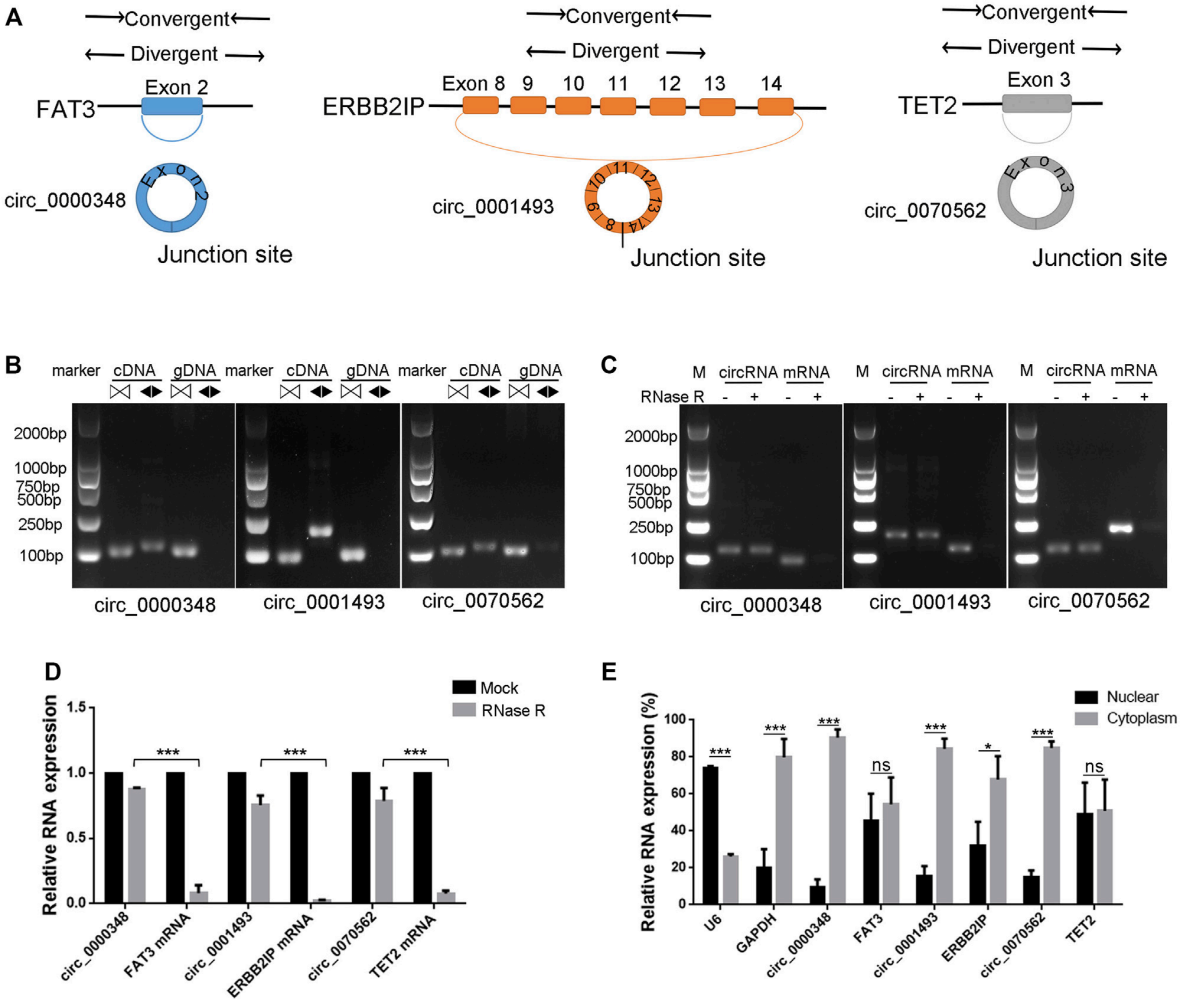
Characterization of hsa_circ_0000348, hsa_circ_0001493 and hsa_circ_0070562 in BMSCs. **(A)**. Schematic illustration showing FAT3 exon two circularisation to form circ_0000348, ERBB2IP exons 8–14 circularisation to form circ_0001493, TET2 exon three circularisation to form circ_0070562. **(B)**. The presence of circ_0000348, circ_0001493 and circ_0070562 were validated in BMSCs by RT-PCR. Divergent primers (◀▶) amplified circRNAsfrom cDNA, but not from genomic 7) DNA. ▷◁ indicates convergent primer. **(C)**. The DNA electrophoresis results of RT-PCR of circ_0000348 and FAT3 mRNA, circ_0001493 and ERBB2IP mRNA, circ_0070562 and TET2 mRNAs in BMSCs treated with or without RNase R. **(D)**. The decrease degree of circRNAs was significantly less than that of their parental genes after RNase R treatment. n = 3 BMSCs samples. **(E)**. Nuclear and cytoplasmic RNAs extraction and qPCR detection revealed circ_0000348, circ_0001493 and circ_0070562 were expressed mainly in the cytoplasm. U6 was used as an internal control for nuclear RNA, whereas GAPDH served as the control for cytoplasmic RNA. *n* = 3 BMSCs samples. Data are presented as the means ± SEM. *p < 0.05. ***p < 0.001. ns: not significant.

The junction sites confirmed circRNAs of circ_0000348, circ_0070562 and circ_0001493 were subjected for further identification. Circularization of FAT Atypical Cadherin 3 (FAT3) exon 2, Erbb2 Interacting Protein (ERBB2IP) exons 8–14, Tet Methylcytosine Dioxygenase 2 (TET2) exon three forms circ_0000348, circ_0001493 and hsa_circ_0070562, respectively (Figure 7A). We designed convergent primers and divergent primers to amplify parental genes and circRNAs using cDNA and genomic DNA (gDNA) (Figure 7A). CircRNAs, including circ_0000348, circ_0001493 and circ_0070562 were amplified by divergent primers in cDNA but not in gDNA (Figure 7B). These three circRNAs were resistant to RNase R, whereas the FAT3, ERBB2IP and TET2 mRNA levels were significantly decreased after RNase R treatment (Figures 7C, D). Lastly, the nuclear and cytoplasmic RNAs were extracted, and junction primers were used for circRNAs detection. U6 was used as an internal control for nuclear RNA, whereas GAPDH served as the control for cytoplasmic RNA. RT-qPCR analysis indicated the abundantly cytoplasmic expression of circ_0000348, circ_0001493 and circ_0070562 in BMSCs (Figure 7E).

### Function of hsa_circ_0000348, hsa_circ_0001493 and hsa_circ_0070562 in Osteogenic Differentiation of AS-BMSCs and HD-BMSCs

**FIGURE 8:**
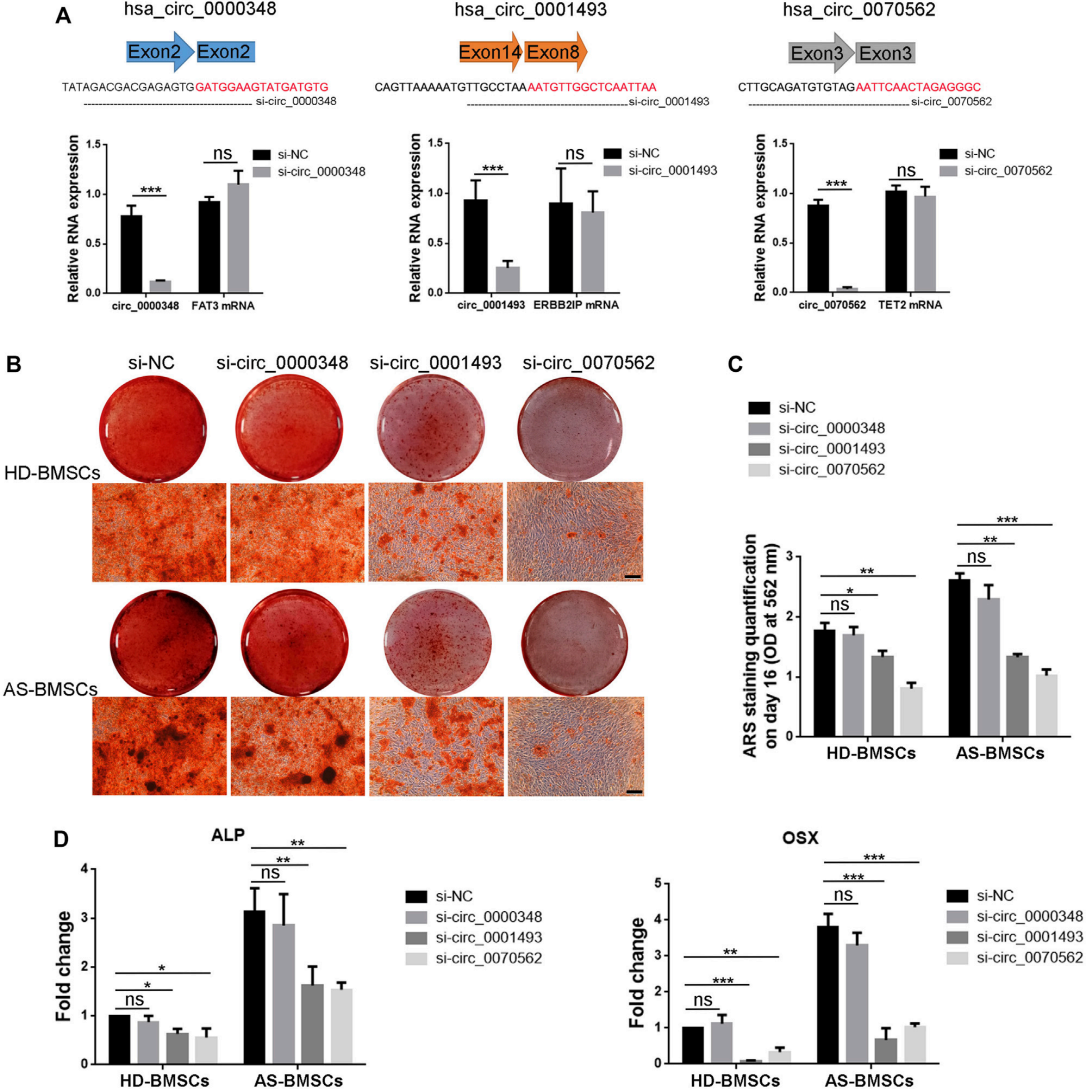
Function of hsa_circ_0000348, hsa_circ_0001493 and hsa_circ_0070562 in osteogenic differentiation of AS-BMSCs and HD-BMSCs. **(A)**. Small interfering RNAs (siRNAs) targeted the junction sites of circ_0000348, circ_0001493 and circ_0070562 were used. qPCR results indicated that the expression of circ_0000348, circ_0001493 and circ_0070562 were decreased, while the expression of FAT3, ERBB2IP and TET2 were not changed in BMSCs transfected with si-circ_0000348, si-circ_0001493, si-circ_0070562 or negative control siRNA (si-NC), respectively, for 48 h at a final concentration of 80 nM. N = 3 BMSCs samples. **(B)**. ARS results of HD-BMSCs and AS-BMSCs transfected si-circ_0000348, si-circ_0001493, si-circ_0070562 or si-NC, respectively, after ostegenic induction for 14 days. Scale bar = 100 μm. **(C)**. The quantitative ARS staining results (Absorbance at 562 nm) in **(B)**. N = 6 BMSCs. **(D)**. qPCR fold change results of ALP and OSX mRNA levels of HD-BMSCs and AS-BMSCs transfected si-circ_0000348, si-circ_0001493, si-circ_0070562 or si-NC, respectively, after ostegenic induction for 3 days. N = 6 BMSCs. *p < 0.05, **p < 0.01, ***p < 0.001.

Next, we tried to demonstrated the role of circ_0000348, circ_0001493 and circ_0070562 in osteogenic differentiation of AS-BMSCs and HD-BMSCs. Small-interfering RNAs (siRNAs) targeting the splicing junction of these three circRNAs were designed respectively, and silencing of the circular transcript significantly reduced the level of circ_0000348, circ_0001493 and circ_0070562, while had no effects on mRNA levels of FAT3, ERBB2IP and TET2, respectively in BMSCs (Figure 8A). The intensity of ARS staining after 14 days’s ostegenic induction of HD-BMSCs and AS-BMSCs transfected with si-circ_0001493 and si-circ_0070562 were both decreased compared to those with si-NC, while it had no obvious differences in the HD-BMSCs and AS-BMSCs transfected with si-circ_0000348, compared to those with si-NC (Figures 8B, C). The knockdown of circ_0001493 and circ_0070562 expression obviously reduced ALP and Osterix (OSX) mRNA levels in HD-BMSCs and AS-BMSCs, as evident from qPCR results (Figure 8D). These data clearly indicated the pro-osteogenic effects of circ_0001493 and circ_0070562 in HD-BMSCs and AS-BMSCs. For the expression of circ_0070562 was increased, while circ_0001493 was decreased in the AS-BMSCs compared with that in the HD-BMSCs verified from the circRNA sequencing and RT-qPCR, circ_0070562 seems to partly mediate the enhanced osteogenically differentiated effect in AS-BMSCs.

## Discussion

In this study, we identified the expression profiles and potential functions of circRNAs in the osteogenically differentiated BMSCs of AS patients, in which 418 circRNAs were DE compared with that in the controls. Among these, 204 circRNAs were upregulated, and 214 were downregulated. In addition, GO and KEGG pathway analyses were performed, and circRNA-miRNA interaction networks were established to help predict the potential functions of the DE circRNAs in new bone formation in AS.

AS is a chronic autoimmune disease. Inflammation and pathological osteogenesis are two main characteristics of AS. Since pathological osteogenesis in AS leads to high disability, intensive studies on the precise mechanisms of pathological osteogenesis in AS patients are required. BMSCs are major sources of osteoblasts, whose normal osteogenic differentiation capacity is of great importance in regulating bone formation *in vivo*, and disruption of this process leads to disease symptoms (Deschaseaux et al., 2010). For example, a deficiency in the osteogenic differentiation capacity of BMSCs contributes to osteoporosis in rheumatoid arthritis (Berthelot et al., 2019). The overactive osteogenic differentiation capacity of BMSCs leads to pathologic osteogenesis in AS, as found by our previous report (Xie et al., 2016a; Xie et al., 2016b) and confirmed by other research teams (Jo et al., 2017; Jo et al., 2018) and this study.

Recently, some molecules that involved in the ossific mechanisms of AS have been demonstrated. For instance, long nocoding RNAs, likely Lnc-ZNF354A-1, lnc-LIN54-1, lnc-FRG2C-3, et al. might involved in the osteogenic differentiation of BMSCs in patients with AS (Xie et al., 2016a). HLA-B27-mediated activation of tissue-nonspecific alkaline phosphatase in BMSCs from enthesis promotes pathogenic syndesmophyte formation in AS (Bolger et al., 2014). Sustained ER stress in AS bone-derived cells stimulates the activation of the RUNX2 and C/EBP-β genes (Jo et al., 2017). Single-nucleotide polymorphism-adjacent super enhancers modulate the enhanced osteogenic differentiation of AS-BMSCs(Yu et al., 2021). However, the function of circRNAs in BMSCs of patients with AS is still unknown and research is needed.

CircRNAs have been shown to function in multiple biological processes, likely protein binding and sequestering, miRNA binding, regulation of transcription and posttranscription, and translation into proteins (Liu et al., 2017; Han et al., 2018; He et al., 2021). In this study, we identified 204 upregulated and 214 downregulated circRNAs. And then GO and KEGG pathway analyses of the host genes of significantly dysregulated circRNAs were conducted. The results of GO analysis suggested that the most significantly enriched GO terms in the biological process, cellular component, and molecular function categories were regulation of cell junction organization, adherens junction, and cadherin binding. Five circRNAs and their parental genes: hsa_circ_0074166/circ_CTNNA1 and CTNNA1, hsa_circ_CTNND1 and CTNND1, hsa_circ_CTTN and CTTN, and hsa_circ_NUMB and NUMB, were enriched in all three GO terms. The results of GO analysis showed that the functions of the circRNA host genes were associated with fundamental pathophysiologic processes essential to AS development. The role of adherens junctions mediated by cadherin in ossification and osteoporosis has been of particular interest (Marie, 2002), and research in this area has greatly expanded over the past few years (Marie et al., 2014; Yang et al., 2020). CTNNA1 and CTNND1 are members of the catenin family of proteins that play an important role in the cell adhesion process and link to the actin filament network, which plays a crucial role in cell differentiation (Jimenez-Caliani et al., 2017). In our study, the expression of hsa_circ_0074166, a circRNA derived from CTNNA1, was significantly downregulated in AS-BMSCs. Hence, hsa_circ_0074166 might be involved in the biological process of adherens junctions by regulating its host gene CTNNA1, which further impacts osteogenesis in AS. Moreover, the results of KEGG analysis revealed that the host genes of significantly dysregulated circRNAs were involved in many important osteogenesis-related pathways, such as the Hippo signaling pathway, Notch signaling pathway, autophagy and TGF-beta signaling pathway. The genes were also enriched in bacterial invasion of epithelial cells and the HIF-1 signaling pathway related to ankylosing spondylitis. In summary, GO and KEGG analyses suggested that the dysregulated circRNAs in osteogenically differentiated BMSCs might be involved in new bone formation in AS. However, further research is needed to confirm these findings.

Another significant role of circRNAs is the sponge adsorption for the miRNAs. In recent years, an increasing number of studies have shown that exonic circRNAs can regulate the expression of genes by adsorbing miRNAs to form a negative regulatory relationship between circRNAs and miRNAs(Yu and Liu, 2019). In our study, we performed miRNA sequencing and predicted the potential miRNA targets of DE circRNAs and constructed a circRNA-miRNA interaction network for the top 20 dysregulated circRNAs. Moreover, Jianqiang Kou et al. investigated changes in circRNA expression profiles in the spinal ligament tissues of patients with AS by RNA-seq and identified 148 DE circRNAs(Kou et al., 2020), of which three circRNAs, circ_0000348, circ_0070562 and circ_0000184, were also found in our DE circRNAs. These three circRNAs-involved circRNA-miRNA networks were predicted. More importantly, the TGF-β signaling pathway, which plays an important role in pathological osteogenesis in AS, was enriched in the DE miRNAs in our sequencing. By combining the DE miRNAs, negative circRNA-miRNA transcriptional relationships associated with the TGF-beta signaling pathway were constructed. Combining the above analysis, the network showed that some of the top 20 dysregulated circRNAs might potentially interact with miRNAs associated with the KEGG pathway TGF-beta, such as circ_0001493, circ_0008126, circ_0002774, circ_0005389 et al. Furthermore, two of the three circRNAs, which were identified in both our and Jianqiang Kou’s sequencing results (Kou et al., 2020): circ_0000348 and circ0070562 were predicted to interact with miR-133b and miR-424–5p that involved in TGF-beta signaling. After qPCR validation, the up-down or down-up pairs of circRNAs and miRNAs, like circ_0001493 with miR-582–3p, circ_0073381 with miR-1291, circ_0000348/circ_0070562 with miR-133b and circ_0070562/circ_0002774 with miR-424–5p were demonstrated, although more studies are still needed to confirm their relationships and roles in enhanced AS-BMSCs osteogenesis. Conclusively, these studies indicated that these dysregulated circRNAs might play important functional roles in the osteogenesis of BMSCs from AS patients by interacting with miRNAs involved in the KEGG pathway TGF-beta. Future studies should focus on these circRNAs and their related miRNAs and mRNAs.

RNA sequencing and the bioinformatic analysis for circRNAs differentially expressed in the AS-BMSCs and HD-BMSCs demonstrated several key circRNAs deserving continued exploration. Circ_0000348, circ_0001493 and circ_0070562 were all involved in the TGF-beta signaling pathway, and circ_0000348 and circ_0070562 were previously reported to be increased in the spinal ligament tissues of patients with AS (Kou et al., 2020) and highly likely participate in AS progress. Circ_0001493 was the mostly down-regulated circRNA in our sequencing (Table 1). So we further inquired into the characterization and function of these three circRNAs. Circ_0000348, circ_0001493 and circ_0070562 were formed by exon(s) circularization and expressed mainly in the cytoplasm. Their back-splice junction sites were confirmed by the Sanger sequencing of qRT-PCR amplified products. Moreover, circ_0070562 mediated the enhanced ostegenesis of AS-BMSCs. Thus, our results demonstrated that circ_0070562 could function as a pro-osteogenic factor in AS, and might serve as a potential biomarker and a therapeutic target for AS diagnosis and treatment. According to our bioinformatic analyses, circ_0070562 could potentially bind to miR-424–5p and miR-133b, which were involved in the KEGG pathways of TGF-beta. miR-424–5p and miR-133b have been reported to attenuate osteogenesis in MSCs (Ru et al., 2020; Wei et al., 2021). Hence, circ_0070562 might enhance AS pathologic osteogenesis progression by regulating miR-424–5p and miR-133b, and their target mRNAs. However, the exact regulatory mechanism of circ_0070562 in ostegenesis of AS-BMSCs is still unknown. Further functional and mechanistic studies of circ_0070562 are needed.

## Conclusion

This study initially revealed the expression profiles of circRNAs in osteogenically differentiated BMSCs from AS patients compared with the healthy donors. By performing high-throughput sequencing, we identified numerous circRNAs were dysregulated in osteogenically differentiated AS-BMSCs compared with that of HD-BMSCs. Bioinformatic analyses indicated that these dysregulated circRNAs might play important functional roles in osteogenesis in AS. The expression of circ_0070562 was increased in AS-BMSCs and associated with enhanced pathological ostegenesis of AS, which might could serve as a potential biomarker and a therapeutic target for AS. However, there are still some limitations of the present study. The novel identified circRNAs should been further explored. And the specific mechanistic study for circ_00070562 participating the pathological osteogenesis in AS is necessary. Further research on circRNAs and circRNA-miRNA interactions involved in AS ossification is imperative.

## Data Availability

The datasets presented in this study can be found in online repositories. The names of the repository/repositories and accession number(s) can be found in the article/Supplementary Material.
