# Supplementary material for: RNA Sequencing Reveals the Expression Profiles of circRNAs and Indicates Hsa_circ_0070562 as a Pro-osteogenic Factor in Bone Marrow-Derived Mesenchymal Stem Cells of Patients With Ankylosing Spondylitis
Source: Front Genet. 2022 Jul 6;13:947120. doi: 10.3389/fgene.2022.947120 (PMC9299369; doi:10.3389/fgene.2022.947120)
Supplement: Supplementary file 2 [file Presentation1.pdf]

# *Supplementary Material*

## 1 Supplemental Methods

### Small RNA sequencing

Total RNA was isolated and the quantity and integrity were assessed as mentioned above. Adapter-ligated RNAs were subjected to RT-PCR and amplified for a low cycle number. Then, the PCR products were size selected by PAGE according to the instructions of the NEBNext® Multiplex Small RNA Library Prep Set for Illumina® (Illumina, USA). Following evaluation by Agilent 2200 TapeStation, the libraries were sequenced by HiSeq 2500 (Illumina, USA) with a single-end 50 bp sequence by RiboBio Co., Ltd. (RiboBio, China). After filtering raw reads, mapping reads were obtained by mapping clean reads to the reference genome. miRDeep2 was used to identify known mature miRNAs and predict novel miRNAs. miRNA expression was calculated as reads per million (RPM). Differential expression between two sets of samples was calculated by the edgeR algorithm according to the following criteria: |log2（Fold Change）|≥1 and P-value < 0.05.

### Sequencing data availability

The sequencing data analyzed in this study have been deposited in NCBI’s Gene Expression Omnibus and are accessible through GEO series accession number GSE178514 (https://www.ncbi.nlm.nih.gov/geo/query/acc.cgi?acc=GSE203155).

## 2 Supplementary Figures

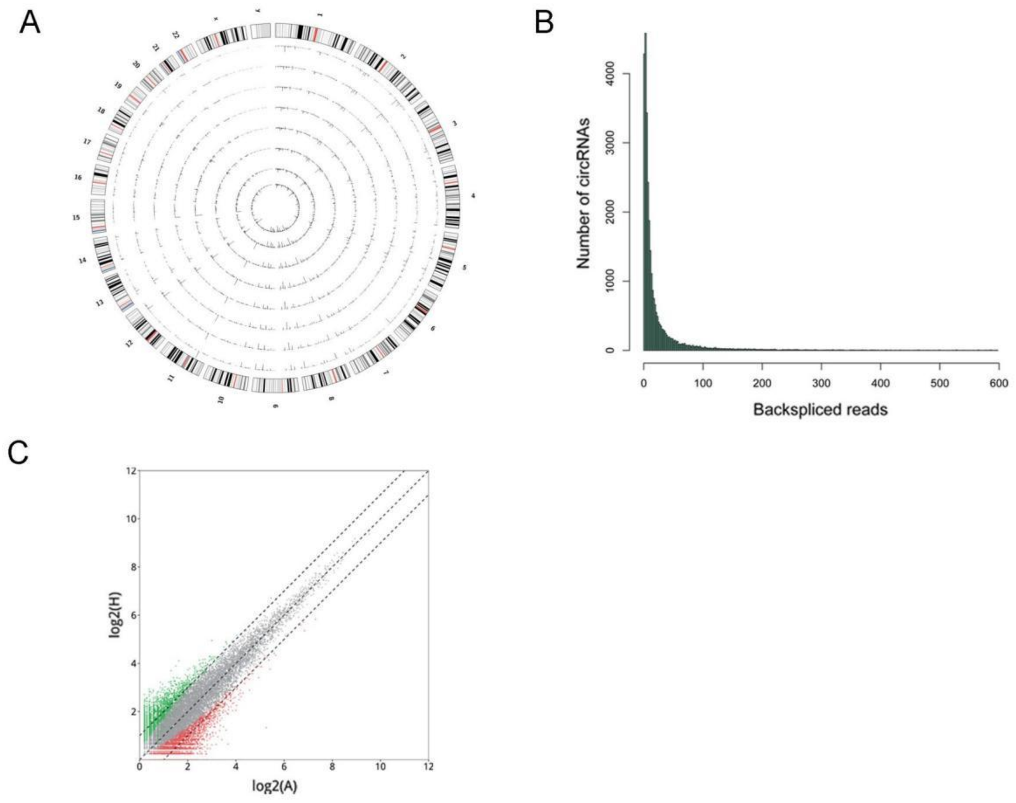


**Figure S1. Characteristics of identified circRNAs by RNA sequencing of HD-BMSCs and AS-BMSCs**

A. Circos plot showing the distribution of identified circRNAs on human chromosomes. The outermost layer is a chromosome map of the human genome. The inner 8 circles represent all circRNAs in each sample detected by RNA-seq. The inner circles from outside to inside corresponded to samples AS1-4 and HD1-4. C. The scatter plot presents the variations in circRNA expression between HD-BMSCs (H) and AS-BMSCs (A). The X and Y axes are the normalized circRNA signal values (log2 scaled). The circRNAs above the top black line and below the bottom black line displayed greater than 2.0-fold upregulation and downregulation, respectively. D. The number of circRNAs in different back-spliced reads.

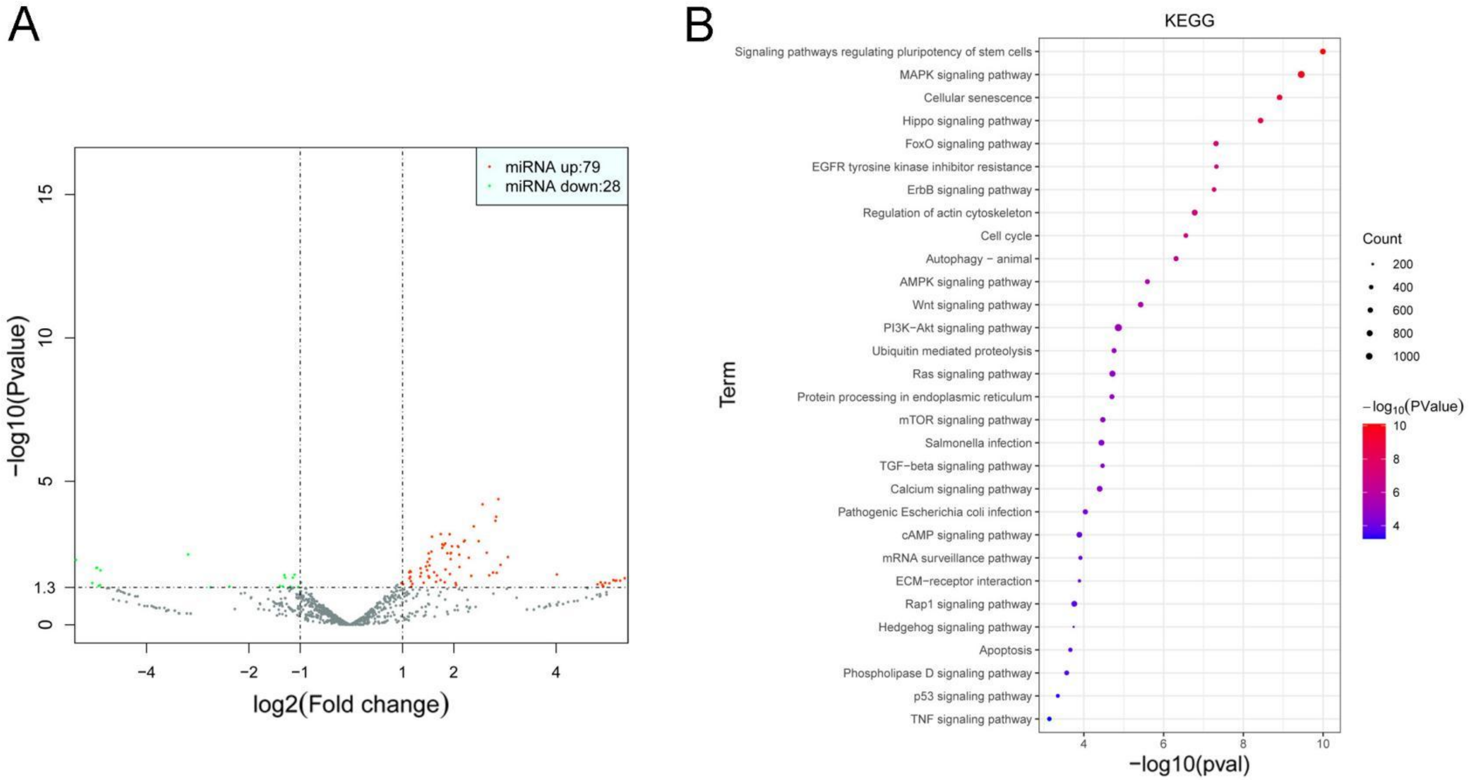


**Figure S2. Differential expression profiles of miRNAs in HD-BMSCs and AS-BMSCs, as determined by RNAseq.**

A. Volcano plots of DE miRNAs in HD-BMSCs and AS-BMSCs. The vertical line indicates a 2.0-fold (log2 scale) change; the horizontal line represents the P value of 0.05 (-log10 scale). Red dots indicate the 79 upregulated miRNAs. Green dots indicate the 28 downregulated miRNAs. B. KEGG analysis revealed that the target genes of DE miRNAs were enriched in osteogenesis-related pathways.

# 3 Supplementary Tables

**Table S1. Primers Used for qPCR in the Validation**

| Gene | Forward Primer (5’-3’) | Reverse Primer (5’-3’) |
|---|---|---|
| GAPDH | GGATGCAGGGATGATGTTC | TGCACCACCAACTGCTTAG |
| U6 | CTCGCTTCGGCAGCACA | AACGCTTCACGAATTTGCGT |
| hsa_circ_0054303 | TTCCGGCAAAACCACATTCA | GGATAGAGTTCTGGGAAACGAG |
| hsa_circ_0073381 | CAGGGAAAGATAACAGGCAACG | GCACGTCCTCCAAAAGAGAGA |
| hsa_circ_0001493 | TGCCAGCAAATTTTGGCAGA | ACATGGTACCAACCGCACAA |
| hsa_circ_0070562 | TTATTGGATACACCTGTCAAGACTC | CTGTTCCATCAGGCTTGCTT |
| hsa_circ_0023231 | ACGGGTTTCTCACAGGATTCTT | TTCAACCGGGTATTCCCCAC |
| hsa_circ_0016408 | ACTGGTTACCTCCCTTTGGGT | TGGTGGTAGCAAAAAGCCAAC |
| hsa_circ_0060150 | AAACAGAACCGACTTCTCCCC | TCCAACGCTTGAAATGGATGA |
| hsa_circ_0005389 | ATCTTCGGCATGTCCTTCGC | GATCACGACGTAGAAGGCGAT |
| hsa_circ_0000348 | GGAAGGTTCAGTATAGACGACGAG | TTGAAAAGCAGGAGGATGAGGC |
| hsa_circ_0039402 | CATGGCTCAACTGGAAGCAC | TTGTCCACATCGTCACAGACA |
| hsa_circ_0059469 | TGCTGGGCTGAAAATACTGGA | GCCCTTCTGCCCTTCACATAA |
| hsa_circ_0002774 | CACGCCCAGTCCGACAAA | TCACTGTAGGTGCTCTCGGA |
| hsa_circ_0062778 | ATGAGGAGAAGGAAAGCCGA | TGAACACTGGGGTCGTAGTC |
| hsa-miR-148a-3p | GTGCACTACAGAACTTTGT | CAGTGCGTGTCGTGGAGT |
| hsa-miR-1291 | TGGCCCTGACTGAAGACCAGCAGT | CAGTGCGTGTCGTGGAGT |
| hsa-miR-582-3p | CTGGTTGAACAACTGAACC | CAGTGCGTGTCGTGGAGT |
| hsa-miR-424-5p | CAGCAGCAATTCATGTTTTGAA | CAGTGCGTGTCGTGGAGT |
| hsa-miR-10b-5p | TACCCTGTAGAACCGAATTTGTG | CAGTGCGTGTCGTGGAGT |
| hsa- miR-340-3p | TCCGTCTCAGTTACTTTATAGC | CAGTGCGTGTCGTGGAGT |
| hsa- miR-133b | TTTGGTCCCCTTCAACCAGCTA | CAGTGCGTGTCGTGGAGT |
| hsa-miR-103a-2-5p | AGCTTCTTTACAGTGCTGCCTTG | CAGTGCGTGTCGTGGAGT |
| hsa-miR-10a-5p | TACCCTGTAGATCCGAATTTGTG | CAGTGCGTGTCGTGGAGT |
| hsa- miR-27a-5p | AGGGCTTAGCTGCTTGTGAGCA | CAGTGCGTGTCGTGGAGT |
| hsa-miR-99a-5p | AACCCGTAGATCCGATCTTGTG | CAGTGCGTGTCGTGGAGT |
| hsa- miR-5006-3p | CCCTTTCCATCCTGGCAG | CAGTGCGTGTCGTGGAGT |
| hsa- miR-671-3p | TCCGGTTCTCAGGGCTCCACC | CAGTGCGTGTCGTGGAGT |
| hsa-miR-548q | GCTGGTGCAAAAGTAATGGCGG | CAGTGCGTGTCGTGGAGT |
| FAT3 | ACTGTATGGGAACAATGGAGTGA | GCTCTTTTTCCAACGAGAAAGGA |
| ERBB2IP | TGTGGCTCTCAGATAATCAGTCC | AGCACCATTTTCTGGGTCTCT |
| TET2 | ATACCCTGTATGAAGGGAAGCC | CTTACCCCGAAGTTACGTCTTTC |
| RUNX2 | TCAACGATCTGAGATTTGTGGG | GGGGAGGATTTGTGAAGACGG |

| | | |
|---|---|---|
| Osterix | CCTCTGCGGGACTCAACAAC | AGCCCATTAGTGCTTGTAAAGG |
| hsa_circ_0000348(Convergent) | TTGTTGCCTCATCCTCCTGC | TGTGTGAAGTGGAAGCCCAG |
| hsa_circ_0001493(Convergent) | TCAATGAAGTTGAAGCTTTGCC | AGTAATTATGATCAGCAGCAAAAGT |
| hsa_circ_0070562(Convergent) | ACCAACCATGTTGAGGGCAA | GGGCTTCCATTCTGGAGCTT |

**Table S2. The Top 10 Terms with the Largest Significant Differences in GO Biological Process Analysis of Parental Genes of DE circRNAs**

| GOBPID | Term | Pvalue | Qvalue | geneNames |
|---|---|---|---|---|
| GO:0034330 | cell junction organization | 0.00000 | 0.00167 | BCAS3/CORO1C/CTNNA1/CTNND1/CTTN/EPHA3/HEG1/MAP4K4/NECTIN3/NF2/NPHP4/NUMB/OPHN1/PARD3/PEAK1/PKN2/PKP4/PTK2/RASSF8/TSC1 |
| GO:0034332 | adherens junction organization | 0.00000 | 0.00350 | BCAS3/CORO1C/CTNNA1/CTNND1/CTTN/EPHA3/MAP4K4/NECTIN3/NUMB/PEAK1/PTK2/RASSF8/TSC1 |
| GO:0007160 | cell-matrix adhesion | 0.00002 | 0.01625 | ADAMTS12/BCAS3/CORO1C/CTTN/EPHA3/MAP4K4/MKLN1/NF2/PEAK1/POSTN/PTK2/SNED1/TRPM7/TSC1/UTRN |
| GO:0034329 | cell junction assembly | 0.00002 | 0.01625 | BCAS3/CORO1C/CTNNA1/CTTN/EPHA3/HEG1/MAP4K4/NPHP4/OPHN1/PARD3/PEAK1/PKN2/PKP4/PTK2/TSC1 |
| GO:0018205 | peptidyl-lysine modification | 0.00005 | 0.03223 | ASH2L/BAG6/CREBBP/CTH/EP300/HDAC4/KAT7/KDM4C/MDM2/NUP98/PHF20/PRDM6/RLF/SENP5/SETD5/SUZ12/TADA2A/TET2/TRPM4/TRRAP |
| GO:0031589 | cell-substrate adhesion | 0.00006 | 0.03407 | ADAMTS12/ARPC2/BCAS3/BVES/CORO1C/CTTN/EPHA3/FBLN1/MAP4K4/MKLN1/NF2/PEAK1/POSTN/PTK2/SNED1/TRPM7/TSC1/UTRN |
| GO:0001952 | regulation of cell-matrix adhesion | 0.00009 | 0.03665 | BCAS3/CORO1C/EPHA3/MAP4K4/NF2/PEAK1/POSTN/PTK2/TSC1/UTRN |
| GO:0048193 | Golgi vesicle transport | 0.00009 | 0.03665 | ACTR1A/ANK3/ANKRD28/ARFGEF2/CAPZB/COG2/COG6/DYNC1H1/ERGIC1/GOLGA3/GOLGB1/KIF2A/LMF1/PPP6R3/SEC24D/STX6/WIPI1 |
| GO:0006914 | autophagy | 0.00013 | 0.03665 | ABL2/ATG3/ATG4B/ATM/ATP6V1H/BNIP3L/CTTN/EP300/IFT88/LARP1/PTK2/SBF2/SH3GLB1/STX12/TBC1D5/TMEM59/TSC1/UBQLN1/USP13/WDFY3/WIPI1/XPA |
| GO:0061919 | process utilizing autophagic mechanism | 0.00013 | 0.03665 | ABL2/ATG3/ATG4B/ATM/ATP6V1H/BNIP3L/CTTN/EP300/IFT88/LARP1/PTK2/SBF2/SH3GLB1/STX12/TBC1D5/TMEM59/TSC1/UBQLN1/USP13/WDFY3/WIPI1/XPA |

**Table S3. The Top 10 Terms with the Largest Significant Differences in GO Cellular Component Analysis of Parental Genes of DE circRNAs**

| GOCCID | Term | Pvalue | Qvalue | geneNames |
|---|---|---|---|---|
| GO:0005912 | adherens junction | 0.00001 | 0.00602 | ARHGAP22/ARHGAP26/ARPC2/CBL/CORO1C/CTNNA1/CTNND1/CTTN/DCAF6/DOCK7/FHL2/G3BP1/LPP/MAP4K4/NF2/NUMB/PDLIM5/PEAK1/PPP1R12A/PTK2/PTPRM/SNTB1/SNTB2/TJP2/TNS3 |
| GO:0030055 | cell-substrate junction | 0.00004 | 0.00957 | ARHGAP22/ARHGAP26/ARPC2/CBL/CORO1C/CTNNA1/CTTN/DCAF6/DOCK7/ERBIN/FHL2/G3BP1/LPP/MAP4K4/NUMB/PEAK1/PPP1R12A/PTK2/SNTB1/SNTB2/TNS3 |
| GO:0030027 | lamellipodium | 0.00009 | 0.00957 | ARPC2/CAPG/CORO1C/CTNNA1/CTNND1/CTTN/MCC/NF2/PKN2/PTK2/PTPRM/SCYL3/TSC1 |
| GO:0005925 | focal adhesion | 0.00010 | 0.00957 | ARHGAP22/ARHGAP26/ARPC2/CBL/CORO1C/CTNNA1/CTTN/DCAF6/DOCK7/FHL2/G3BP1/LPP/MAP4K4/NUMB/PEAK1/PPP1R12A/PTK2/SNTB1/SNTB2/TNS3 |
| GO:0005924 | cell-substrate adherens junction | 0.00010 | 0.00957 | ARHGAP22/ARHGAP26/ARPC2/CBL/CORO1C/CTNNA1/CTTN/DCAF6/DOCK7/FHL2/G3BP1/LPP/MAP4K4/NUMB/PEAK1/PPP1R12A/PTK2/SNTB1/SNTB2/TNS3 |
| GO:0005813 | centrosome | 0.00036 | 0.02569 | ACTR1A/ARHGEF10/C2CD3/CDK5RAP2/CEP85L/CLIP1/CNTLN/CTDP1/DYNC1H1/ERC1/IFT88/KIF2A/MAP3K11/MASTL/NEK6/NPHP4/PKN2/PPP1R12A/RAB11FIP3/SDCCAG8/TNKS2 |
| GO:0030426 | growth cone | 0.00039 | 0.02569 | CBL/CTNND1/CTTN/DOCK7/EXOC6/KIF5B/PARD3/PTBP2/TRAK1/TSC1/UTRN |
| GO:0030427 | site of polarized growth | 0.00045 | 0.02617 | CBL/CTNND1/CTTN/DOCK7/EXOC6/KIF5B/PARD3/PTBP2/TRAK1/TSC1/UTRN |
| GO:0017119 | Golgi transport complex | 0.00104 | 0.04867 | COG2/COG6/GOLGA3 |
| GO:0030122 | AP-2 adaptor complex | 0.00104 | 0.04867 | AP2A2/PICALM/TBC1D5 |

**Table S4. The Top 10 Terms with the Largest Significant Differences in GO Molecular Function of Parental Genes of DE circRNAs**

| GOMFID | Term | Pvalue | Qvalue | geneNames |
|---|---|---|---|---|
| GO:0045296 | cadherin binding | 0.00000 | 0.00055 | ANK3/CAPG/CAPZB/CBL/CTNNA1/CTNND1/CTTN/ERC1/GOLGA3/KIF5B/LARP1/NUMB/PDLIM5/PFKP/PICALM/PKN2/PTPRM/RARS/SH3GLB1/TJP2/UBAP2 |
| GO:0050839 | cell adhesion molecule binding | 0.00005 | 0.00984 | ANK3/CAPG/CAPZB/CBL/CTNNA1/CTNND1/CTTN/ERC1/GOLGA3/KIF5B/LARP1/NECTIN3/NUMB/PDLIM5/PFKP/PICALM/PKN2/PTPRM/RARS/SH3GLB1/TJP2/UBAP2/UTRN |
| GO:0035257 | nuclear hormone receptor binding | 0.00005 | 0.00984 | BAZ2A/BCAS3/CNOT1/EP300/FHL2/KDM4C/LATS1/MED17/MED25/NCOR2/PRPF6/WIPI1 |
| GO:0051427 | hormone receptor binding | 0.00022 | 0.02953 | BAZ2A/BCAS3/CNOT1/EP300/FHL2/KDM4C/LATS1/MED17/MED25/NCOR2/PRPF6/WIPI1 |
| GO:0033613 | activating transcription factor binding | 0.00034 | 0.03677 | CREBBP/DHX33/EP300/HDAC4/NEK6/TP53BP1/YAP1 |
| GO:0035258 | steroid hormone receptor binding | 0.00046 | 0.04190 | CNOT1/EP300/FHL2/KDM4C/LATS1/NCOR2/PRPF6/WIPI1 |
| GO:0017016 | Ras GTPase binding | 0.00104 | 0.08084 | ACAP2/ANKRD27/ARHGEF10/ARHGEF28/CORO1C/DENND5A/DOCK4/DOCK7/ERC1/IPO11/MAP3K11/PICALM/PKN2/RAB11FIP3/SBF2/TNPO1 |
| GO:0031267 | small GTPase binding | 0.00157 | 0.10679 | ACAP2/ANKRD27/ARHGEF10/ARHGEF28/CORO1C/DENND5A/DOCK4/DOCK7/ERC1/IPO11/MAP3K11/PICALM/PKN2/RAB11FIP3/SBF2/TNPO1 |
| GO:0050681 | androgen receptor binding | 0.00204 | 0.12283 | EP300/FHL2/KDM4C/PRPF6/WIPI1 |
| GO:0003712 | transcription coregulator activity | 0.00332 | 0.17350 | ATF6/CASP8AP2/CREBBP/DCAF6/EP300/FHL2/GON4L/HDAC4/MED17/N4BP2L2/NCOR2/NUP98/PER2/PRPF6/SS18/TBPL1/TP53BP1/TRRAP/YAP1/ZMYND8 |
